# Hemodynamic and Lesion Characteristics Associated with Discordance between the Instantaneous Wave-Free Ratio and Fractional Flow Reserve

**DOI:** 10.1155/2019/3765282

**Published:** 2019-07-14

**Authors:** Hiroyuki Arashi, Natsuko Satomi, Issei Ishida, Kanintorn Soontorndhada, Suguru Ebihara, Kazuki Tanaka, Hisao Otsuki, Masashi Nakao, Kentaro Jujo, Junichi Yamaguchi, Nobuhisa Hagiwara

**Affiliations:** Department of Cardiology, The Heart Institute of Japan, Tokyo Women's Medical University, Tokyo, Japan

## Abstract

**Background:**

The instantaneous wave-free ratio (iFR) is an invasive coronary physiological index that is not inferior to fractional flow reserve- (FFR-) guided revascularization. The indexes of iFR and FFR are similar and closely correlated, but there are a few key differences. Previous studies suggested that patient characteristics and lesion severity could induce discordance between iFR and FFR. This study aimed to clarify the hemodynamics and lesion characteristics that influence discordance between iFR and FFR.

**Methods:**

In this retrospective study, we enrolled 225 patients (304 lesions) who underwent clinically indicated invasive coronary angiography and both iFR and FFR examinations between 2012 and 2017. We included only patients who underwent right heart catheterization and had blood pressure and heart rates recorded immediately prior to iFR and FFR.

**Results:**

Discordance (iFR ≤0.89 and FFR >0.8 or iFR >0.89 and FFR ≤0.8) was observed in 80 lesions (26.3%). The heart rate, rate-pressure product, and cardiac index tended to be higher in the iFR ≤0.89 group than in the iFR >0.89 group. These trends were not seen between the FFR ≤0.8 group and FFR >0.8 group. A multivariate analysis showed that independent predictors of iFR ≤0.89 and FFR >0.8 discordance were female sex and higher rate-pressure product. iFR >0.89 and FFR ≤0.8 discordance was rare in hemodialysis patients.

**Conclusion:**

Even if iFR is functionally significant in intermediate stenosis, additional FFR evaluations should be considered for women, especially those with a high rate-pressure product, to avoid unnecessary percutaneous coronary intervention. If iFR is not functionally significant with intermediate stenosis in hemodialysis patients, then further FFR evaluations are unnecessary.

## 1. Introduction

Percutaneous coronary intervention (PCI) performed under the guidance of fractional flow reserve (FFR) has been associated with significantly fewer adverse events than PCI performed under angiographic guidance [1–3]. The instantaneous wave-free ratio (iFR) is another invasive coronary physiological index that has been shown to be not inferior to FFR-guided revascularization. Randomized controlled trials have suggested a cutoff point of iFR ≤0.89 for the iFR index [4, 5]. Although FFR ≤0.8 or iFR ≤0.89 can identify ischemia-inducing coronary stenoses with high accuracy, these two indices can sometimes show discordance in the clinical setting [6, 7]. Furthermore, several studies have reported that left ventricular filling pressure and hemodialysis therapy could influence the relationship between iFR and FFR [8, 9]. The effects of hemodynamic status and lesion characteristics on discordance between iFR and FFR have not been thoroughly evaluated. Therefore, the purpose of this study was to clarify the factors of hemodynamics and lesion characteristics that influence discordance between iFR and FFR.

## 2. Materials and Methods

### 2.1. Patient Population

In this retrospective study, we enrolled patients who underwent clinically indicated invasive coronary angiography as well as both iFR and FFR examinations between 2012 and 2017. Because we defined the hemodynamic status using the rate-pressure product and right heart catheterization parameters, we limited the analysis to patients who underwent right heart catheterizations and had blood pressure and heart rates measured just before the iFR and FFR. All patients had at least one intermediate lesion with stenosis with a diameter >25% on quantitative coronary angiography. We excluded lesions in the left main trunk and those in bypass grafts. Patients with ST-segment elevation myocardial infarction, non–ST-segment elevation myocardial infarction, or New York Heart Association class IV heart failure were excluded. In the present study, the discordance of iFR and FFR was defined either as iFR ≤0.89 and FFR >0.8 or as iFR >0.89 and FFR ≤0.8.

### 2.2. Hemodynamic Parameters and Right Heart Catheterization Measurements

Right heart catheterization was performed using a flow-guided Swan–Ganz thermodilution catheter via the brachial, internal jugular, or femoral veins. Hemodynamic parameters including central venous pressure, pulmonary artery wedge pressure, and cardiac index were measured just before coronary angiography. Thermodilution cardiac output was determined by injecting 10 mL of ice-cold saline in the right atrium. The average of several consecutive measurements with less than 10% variation was calculated. The rate-pressure product was defined as the systolic blood pressure multiplied by the heart rate (both were measured just before iFR measurements). Standard 12-lead electrocardiograms were also performed using the interpretive ECG-1550 (Nihon Kohden, Tokyo, Japan). RV5 plus SV1, which has been associated with left ventricular hypertrophy, was calculated [10]. All parameters were compared between the concordant group and discordant group.

### 2.3. Coronary Angiography and Quantitative Coronary Angiography

Coronary angiography was performed according to standard clinical methods via the radial or femoral arterial approach. Quantitative coronary angiography was performed by an independent physician using a computer-assisted automated edge detection algorithm (AWOS; Siemens, Munich, Germany); the physician was blinded to the results of the iFR and FFR. The external diameter of the contrast-filled catheter (5 Fr or 6 Fr) was used as the calibration standard. The percentage of the stenosis diameter during end-diastole was measured using the worst-view trace. Lesion lengths were measured as the distance between the proximal and distal shoulders in the projection demonstrating stenosis with the least amount of foreshortening.

### 2.4. Standard iFR and FFR Measurements

Both iFR and FFR examinations were performed using either diagnostic or interventional guiding catheters. After administration of an intracoronary bolus of nitroglycerin, a pressure wire (Prime Wire Prestige; Philips Volcano Corporation, San Diego, CA) was advanced to the tip of the catheter; the pressure was equalized against that measured through the guiding catheter. After pressure equalization at the tip of the guide catheter had been completed, the guidewire was advanced to a point distal to the stenosis. First, iFR was directly and automatically measured online using the Volcano Core system (Philips Volcano). Second, the FFR was measured during maximal hyperemia. Hyperemia in the target coronary artery was achieved either with an intracoronary bolus injection of 8-12 mg papaverine or with continuous intravenous administration of adenosine at 150 *μ*g/kg/min. At the end of each measurement, the pressure sensor was retracted to the tip of the guide catheter to avoid pressure drift.

### 2.5. Statistical Analysis

Normally distributed data are reported as means ± standard deviations (SD). Categorical data are reported as absolute values and percentages. Depending on the data characteristics, the four groups were compared using an analysis of variance, the Kruskal-Wallis test, or the chi-square test. Correlations between parameters were tested using Pearson's or Spearman's correlation coefficients. A multiple logistic regression model that included factors identified as potentially significant (p<0.1) in the univariate analysis was used. P value of <0.05 was considered to indicate statistical significance. Statistical analyses were performed using statistical software (JMP Pro 14.0; SAS Institute Inc., Cary, NC).

### 2.6. Compliance with Ethical Standards

The study protocol was performed based on the regulations of the hospital's ethics committee. All participating patients provided written informed consent. The study was conducted according to the principles of the Declaration of Helsinki.

## 3. Results

**Figure 1:**
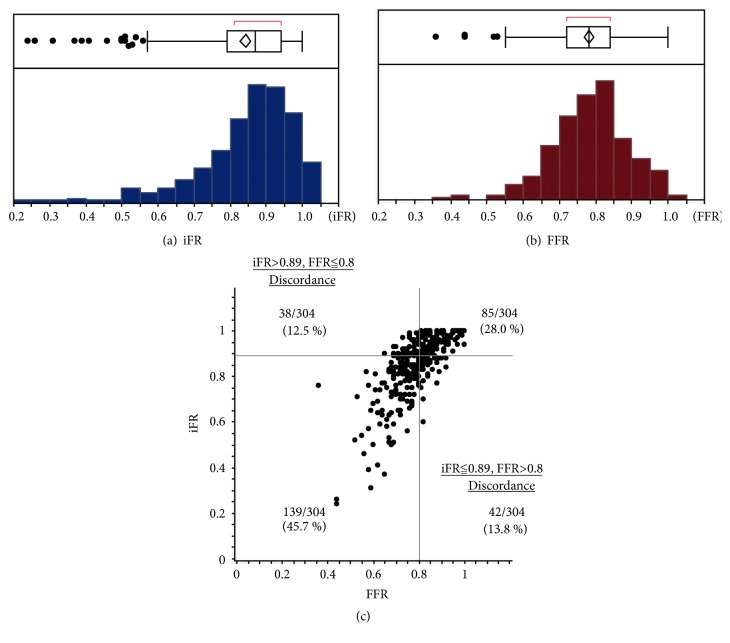
Distribution of the iFR (a) and FFR (b), and the scatter plot comparing the iFR and FFR (c). Discordance of iFR ≤0.89 and FFR >0.8 was observed in 42 (13.8%) lesions. Discordance of iFR >0.89 and FFR ≤0.8 occurred in 38 (12.5%) lesions. iFR: instantaneous wave-free ratio; FFR: fractional flow reserve.

We enrolled 304 lesions from 225 consecutive patients in this study. The study patient characteristics are shown in Table 1. The mean patient age was 66.8 ± 10.7 years, 59.1% had diabetes mellitus, 75.6% had hypertension, and 67.1% had hypercholesterolemia. The prevalence of hemodialysis was 27.1%, and the mean left ventricular ejection fraction was 49.9%. The distribution of iFR and FFR for lesions as well as the correlation between iFR and FFR is shown in Figure 1. The iFR was significantly and positively correlated with FFR (Pearson's correlation: r=0.72; p < 0.0001). The discordance of iFR ≤0.89 and FFR >0.8 was observed in 42 (13.8%) lesions, whereas the discordance of iFR >0.89 and FFR ≤0.8 was observed in 38 (12.5%) lesions (Figure 1(c)).  Table 2 shows the lesion characteristics of the concordant and discordant groups. The discordance of iFR ≤0.89 and FFR >0.8 was present significantly more often in women. The discordance of iFR >0.89 and FFR ≤0.8 was less frequently observed in patients with diabetes mellitus and those on hemodialysis. The glomerular filtration rate of patients in the discordance of iFR >0.89 and FFR ≤0.8 group was higher than that of patients in the other group. The hemodynamic and angiographic parameters for iFR and FFR in the concordant and discordant groups are shown in Table 3. The heart rate and rate-pressure product of the iFR ≤0.89 group were significantly higher than those of the iFR >0.89 group. Regarding right heart catheter data, the cardiac index of the iFR ≤0.89 group was significantly higher than that of the iFR >0.89 group. No difference was observed with hemodynamic parameters between the FFR ≤0.8 group and FFR >0.8 group (Table I in the Data Supplement). Left anterior descending artery lesions and long lesion lengths tended to be associated with iFR ≤0.89 and FFR ≤0.8 concordance. In contrast, lesions located in arteries other than the left anterior descending artery, those with a smaller percentage of stenosis diameter values, those with higher reference diameters, and those with shorter lesion lengths tended to be associated with iFR >0.89 and FFR >0.8 concordance.

Female sex (P=0.01), current smoking status (P=0.05), high rate-pressure product (P=0.02), and short lesion length (P=0.06) were potentially associated with discordance of iFR ≤0.89 and FFR >0.8 in the univariate analysis. The prevalence of diabetes mellitus (P=0.06), not being on hemodialysis (P=0.005), low rate-pressure product (P=0.02), and low cardiac index values (P=0.01) were potentially associated with discordance of iFR >0.89 and FFR ≤0.8 in the univariate analysis. The multivariate analysis showed that the independent predictors for discordance of iFR ≤0.89 and FFR >0.8 were female sex (hazard ratio [HR], 2.84; 95% confidence interval [CI], 1.36-5.96; p=0.01) and high rate-pressure product (HR, 1.17; 95% CI, 1.02-1.34; p=0.03) (Table 4(a)). Furthermore, the status of not being on hemodialysis was independently associated with discordance of iFR >0.89 and FFR ≤0.8 (HR, 0.07; 95% CI, 0.01-0.53; p=0.01) (Table 4(b)).

**Figure 2:**
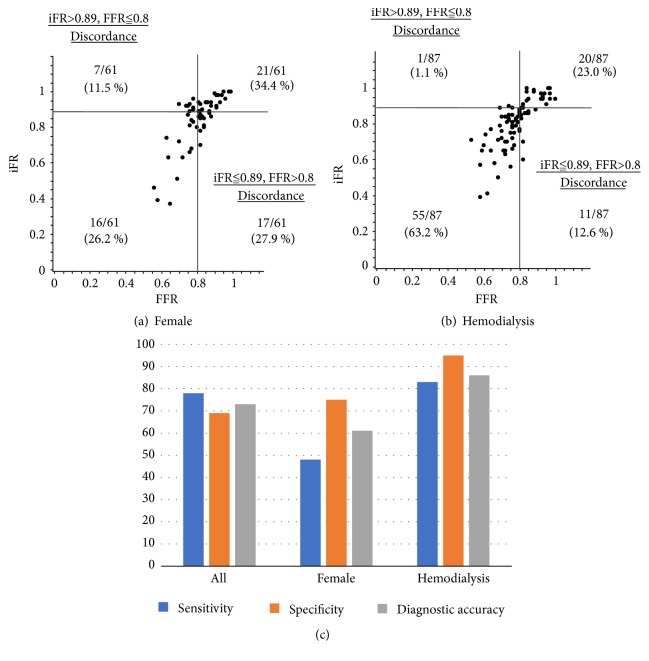
Scatter plots of the iFR and FFR among women (a) and hemodialysis patients (b). Sensitivity, specificity, and diagnostic accuracy of iFR among the entire study population, women, and hemodialysis patients (c). Among women, diagnostic sensitivity was lower than that for the entire population (48% vs 78%, respectively). In contrast, the diagnostic specificity among hemodialysis patients was higher than that for the entire population (95% vs 69%, respectively). iFR: instantaneous wave-free ratio; FFR: fractional flow reserve.

The scatterplots showing the relationship between iFR and FFR for women and hemodialysis patients are shown in Figure 2. Among women, the discordance of iFR ≤0.89 and FFR >0.8 was seen in 27.9% (Figure 2(a)). Among hemodialysis patients, the discordance of iFR >0.89 and FFR ≤0.8 was scarce (Figure 2(b)).  Figure 2(c) shows diagnostic sensitivities, specificities, and accuracies of iFR when FFR was used as the gold standard for the entire study population, women, and hemodialysis patients. Diagnostic sensitivity was lower for women than for the entire study population (48% vs 78%, respectively). The diagnostic specificity for hemodialysis patients was higher than that for the entire study population (95% vs 69%, respectively).

## 4. Discussion

The present study resulted in three primary findings. First, 26.3% of the discordance between iFR and FFR occurred in cases of intermediate coronary artery stenosis. Second, the high rate-pressure product and female sex were independent predictors of discordance of iFR ≤0.89 and FFR >0.8. Third, the discordance of iFR >0.89 and FFR ≤0.8 rarely occurred in hemodialysis patients.

### 4.1. Influence of the Hemodynamic Factor on iFR and FFR Discordance

The pressure wire-derived indexes of iFR and FFR are similar because both are calculated by comparing the coronary artery pressure to the aortic pressure. However, these two indexes differ in several important ways. The FFR is calculated under hyperemic conditions during the entire cardiac cycle. In contrast, the iFR is calculated under resting conditions during a specific period in the diastole.

In our study, heart rate, rate-pressure product, and cardiac index tended to be higher in the iFR ≤0.89 group than in the iFR >0.89 group. These trends were not seen between the FFR ≤0.8 group and FFR >0.8 group. Although there was no significant correlation between the rate-pressure product and FFR, the rate-pressure product and iFR showed a weak, but significant, negative correlation (Pearson's correlation: r=0.15; p=0.01) (Figure I in the Data Supplement). Additionally, the correlation between the cardiac index and iFR was better than the correlation between the cardiac index and FFR (Figure II in the Data Supplement). These data suggest that iFR values might be influenced by hemodynamic factors. In contrast, FFR values were independent of the hemodynamic factors.

### 4.2. Factors Influencing Discordance of iFR ≤0.89 and FFR >0.8

The multivariate analysis revealed that female sex was an independent predictor of discordance of iFR ≤0.89 and FFR >0.8. Similarly, Lee et al. reported that female sex was significantly associated with low iFR and high FFR [6]. The discordance of iFR ≤0.89 and FFR >0.8 might be caused by either an overestimation of iFR or an underestimation of FFR (or both). It is well known that women have a lower coronary flow reserve than men with nonobstructive coronary artery disease [11]. Kobayashi et al. reported that the reduction in the coronary flow reserve of women is not due to reduced hyperemic flow; instead, it is due to the higher resting coronary flow [12]. In contrast, it has been reported that FFR and iFR discordance could be explained by differences in hyperemic coronary flow [13]. The exact mechanism of discordance of iFR ≤0.89 and FFR >0.8 is unclear. We also found that the high rate-pressure product was significantly associated with discordance of iFR ≤0.89 and FFR >0.8. The myocardial blood flow correlated linearly with rate-pressure products under resting conditions. In contrast, hyperemic blood flow was no longer correlated with rate-pressure products [14]. Higher baseline coronary flow induces larger translesional pressure losses and may result in lower iFR values. Therefore, it is tempting to speculate that high coronary baseline flow in women and high rate-pressure products might be mechanisms of discordance between iFR and FFR.

### 4.3. Factors Influencing Discordance of iFR >0.89 and FFR ≤0.8

In our study, the multivariate analysis revealed that not being on hemodialysis was an independent predictor of iFR >0.89 and FFR ≤0.8 discordance. This means that discordance of iFR >0.89 and FFR ≤0.8 occurred rarely in hemodialysis patients. Morioka et al. reported that the iFR values tended to be lower in hemodialysis patients than in nonhemodialysis patients [9]. Hemodialysis patients have higher baseline coronary flow, and iFR values obtained under submaximal hyperemia conditions could be similar to FFR values.

When the aforementioned multivariate analysis of the factors influencing discordance between iFR and FFR was limited to lesions with 40-80% stenosis, which are commonly accepted as intermediate stenosis, female sex was an independent predictor of discordance of iFR ≤0.89 and FFR >0.8. Furthermore, not being on hemodialysis was an independent predictor of iFR >0.89 and FFR ≤0.8 discordance. Interestingly, the rate-pressure product was not a predictor of iFR ≤0.89 and FFR >0.8 discordance, but rather a predictor of iFR >0.89 and FFR ≤0.8 discordance (Table II in the Data Supplement). These results were similar to those reported by Derimay et al. [7]. Those results reinforced that female sex, the rate-pressure product, and being on hemodialysis are related to iFR and FFR discordance.

### 4.4. Diagnostic Performance and Clinical Implications

The iFR is advantageous because of its shorter procedure time, lower cost, and fewer postprocedure complications compared to FFR. The merits of FFR include its reproducibility and stability because it is measured under hyperemic conditions. In our study, when setting FFR as the gold standard, the sensitivity of iFR among women was lower and the specificity of iFR among hemodialysis patients was higher than those of our overall study population. When evaluating coronary artery stenosis with iFR, there is a tendency for women to have more false-positive results, and false-negative results are extremely rare for hemodialysis patients. Even if the iFR is functionally significant for intermediate stenosis, an additional FFR evaluation should be considered for female patients, especially those with high rate-pressure product, to avoid unnecessary PCI. If the iFR is not functionally significant for intermediate stenosis in hemodialysis patients, then further FFR evaluations are not necessary. Additional studies with a prospective study design and larger numbers of patients are necessary to validate the current findings.

### 4.5. Study Limitations

This study had some limitations. First, this was a retrospective observational cohort study conducted at a single center, and the number of study patients was relatively small. Second, discordance was observed in 26.3% of cases in this study, which was relatively higher than that observed in previous studies. Third, the prevalence rates of diabetes mellitus, hypertension, and hemodialysis were higher than those of previous studies. These comorbidities induce structural changes in the myocardium and reduce coronary capacity, which might influence the relationship between iFR and FFR. Furthermore, hemodialysis patients characteristically exhibit left ventricular hypertrophy, reduced arterial compliance, and impaired microcirculation, and the time from last dialysis to catheterization affected the results; therefore, we could not exclude the possibility of bias.

## 5. Conclusion

Even if the iFR is functionally significant for intermediate stenosis, additional FFR evaluations should be considered for women, especially those with high rate-pressure product, to avoid unnecessary PCI. If the iFR is not functionally significant in cases of intermediate stenosis in hemodialysis patients, then further FFR evaluations are not necessary.

## Figures and Tables

**Table 1:** Baseline patient characteristics.

Variable	n = 225
Age, years	66.8 ± 10.7
Men, n (%)	179 (79.6)
Body mass index, kg/m^2^	23.9 ± 4.1
Diabetes mellitus, n (%)	133 (59.1)
Hypertension, n (%)	170 (75.6)
Hypercholesterolemia, n (%)	151 (67.1)
Smoking, n (%)	95 (42.2)
Previous myocardial infarction, n (%)	54 (24.0)
Previous PCI/CABG, n (%)	118 (52.4)
LVEF (%)	49.9 ± 11.1
GFR, mL/min/1.73 m^2^	44.9 ± 27.6
Hemodialysis, n (%)	61 (27.1)

PCI, percutaneous coronary intervention; CABG, coronary aorta bypass grafting; LVEF, left ventricular ejection fraction; GFR, glomerular filtration rate.

Data are expressed as number (percentage) or mean ± SD.

**Table 2:** Lesion characteristics of the concordant and discordant groups.

	iFR ≤0.89 FFR >0.8 Discordance (n = 42)	iFR ≤0.89 FFR ≤0.8 Both positive (n = 139)	iFR >0.89 FFR >0.8 Both negative (n = 85)	iFR >0.89 FFR ≤0.8 Discordance (n = 38)	p-value
Age	68.7 ± 11.6	66.0 ± 10.4	69.2 ± 10.5	66.0 ± 11.0	0.11
Female sex	17 (40.5 %)	16 (11.5%)	21 (24.7 %)	7 (18.4 %)	0.001
Body mass index	23.4 ± 3.8	24.0 ± 5.1	23.1 ± 3.5	24.0 ± 3.4	0.45
Diabetes mellitus, n (%)	28 (66.7 %)	96 (69.1 %)	44 (51.8 %)	18 (47.3 %)	0.02
Hypertension, n (%)	33 (78.6 %)	108 (77.7 %)	65 (76.5 %)	27 (71.1 %)	0.84
Dyslipidemia, n (%)	30 (71.4 %)	93 (66.9 %)	58 (68.2 %)	29 (76.3 %)	0.70
Smoking, n (%)	12 (28.6 %)	61 (43.9 %)	39 (45.8%)	17 (44.7 %)	0.25
Previous myocardial infarction, n (%)	9 (21.4%)	31 (22.3 %)	28 (32.9%)	6 (15.8 %)	0.15
Revascularization, n (%)	19 (45.2 %)	71 (51.1 %)	54 (63.5 %)	21 (55.3 %)	0.18
LVEF, %	49.2 ± 11.6	48.8 ± 10.9	49.7 ± 12.1	51.5 ± 9.6	0.63
GFR, mL/min/1.73 m^2^	40.3 ± 27.8	37.3 ± 28.0	48.5 ± 27.5	60.0 ± 18.8	<.0001
Hemodialysis, n (%)	11 (26.2%)	55 (39.6 %)	20 (23.5%)	1 (2.6%)	<.0001

iFR, instantaneous wave-free ratio; FFR, fractional flow reserve; LVEF, left ventricular ejection fraction; GFR, glomerular filtration rate.

Data are expressed as number (percentage) or mean ± SD.

**Table 3:** Hemodynamic and lesion factors for iFR and FFR in the concordant and discordant groups.

	iFR ≤0.89 FFR >0.8 Discordance (n = 42)	iFR ≤0.89 FFR ≤0.8 Both positive (n = 139)	iFR >0.89 FFR >0.8 Both negative (n = 85)	iFR >0.89 FFR ≤0.8 Discordance (n = 38)	p-value
Systolic blood pressure, mmHg	129 ± 22.3	126 ± 23.4	124 ± 26.2	122 ± 22.5	0.52
Diastolic blood pressure, mmHg	62.2 ± 9.6	60.5 ± 13.1	63.6 ± 14.3	65.0 ± 13.9	0.17
Heart rate, beat/min	75.9 ± 14.6	72.9 ± 11.7	69.1 ± 11.9	67.1 ± 11.7	0.002
Rate-pressure product	9822 ± 2660	9186 ± 2367	8604 ± 2311	8166 ± 2062	0.001
CVP, mmHg	7.2 ± 2.6	6.6 ± 3.1	6.4 ± 3.1	6.9 ± 3.2	0.5
PAWP, mmHg	13.4 ± 4.4	13.2 ± 5.9	12.3 ± 4.9	12.0 ± 4.7	0.37
Cardiac index, L/min/m^2^	3.4 ± 0.9	3.4 ± 1.2	3.1 ± 0.9	2.9 ± 0.6	0.01
RV5 + SV1, mV	3.1 ± 1.2	2.9 ± 1.3	2.7 ± 1.5	2.5 ± 1.8	0.27
Lesion location					
LAD, n (%)	21 (50.0)	99 (71.2)	24 (28.2)	22 (57.9)	<.0001
Proximal lesion	14 (33.3)	62 (44.6)	31 (36.5)	17 (44.7)	0.44
Tandem/diffuse lesion	10 (23.8)	51 (36.7)	18 (21.2)	9 (23.7)	0.055
Diameter stenosis, %	59.5 ± 18.3	65.3 ± 17.8	58.2 ± 16.6	65.4 ± 17.3	0.01
Reference diameter, mm	2.71 ± 0.41	2.62 ± 0.44	2.9 ± 0.55	2.7 ± 0.64	0.001
Lesion length, mm	16.2 ± 5.2	20.4 ± 8.4	14.9 ± 4.8	18.6 ± 5.5	<.0001

iFR, instantaneous wave-free ratio; FFR, fractional flow reserve; CVP, central vein pressure; PAWP, pulmonary artery wedge pressure; LAD, left anterior descending artery.

Data are expressed as number (percentage) or mean ± SD.

**(a):** 

	Discordance of iFR ≤0.89 and FFR >0.8					
	Univariate analysis			Multivariate analysis		
	Odds ratio	95% CI	p-value	Odds ratio	95% CI	p-value
Age	1.01*∗*	0.98-1.04	0.34			
Female sex	3.4	1.68-6.76	0.01	2.84	1.36-5.96	0.01
Diabetes mellitus	1.32	0.66-2.62	0.43			
Hypertension	1.14	0.52-2.51	0.75			
Hypercholesterolemia	1.14	0.55-2.34	0.72			
Hemodialysis	0.87	0.42-1.82	0.71			
Smoking	0.49	0.24-1.01	0.05	0.64	0.30-1.37	0.24
Rate-pressure products	1.17‡	1.03-1.33	0.02	1.17‡	1.02-1.34	0.03
Cardiac index	1.10*∗*	1.82-1.47	0.52			
Lesion located in LAD	0.81	0.42-1.55	0.52			
Diameter stenosis	0.89†	0.74-1.08	0.23			
Reference diameter	0.92*∗*	0.49-1.75	0.80			
Lesion length	0.62†	0.37-1.01	0.06	0.57†	0.32-1.02	0.06

**(b):** 

	Discordance of iFR >0.89 and FFR ≤0.8					
	Univariate analysis			Multivariate analysis		
	Odds ratio	95% CI	p-value	Odds ratio	95% CI	p-value
Age	0.98*∗*	0.95-1.02	0.44			
Female sex	0.89	0.37-2.12	0.78			
Diabetes mellitus	0.50	0.26-1.04	0.06	0.57	0.28-1.16	0.12
Hypertension	0.71	0.33-1.53	0.39			
Hypercholesterolemia	1.51	0.69-3.34	0.30			
Hemodialysis	0.06	0.01-0.42	0.005	0.07	0.01-0.53	0.01
Smoking	1.11	0.56-2.20	0.44			
Rate-pressure products	0.83‡	0.71-0.98	0.02	0.88‡	0.74-1.04	0.13
Cardiac index	0.57*∗*	0.35-0.92	0.01	0.80*∗*	0.48-1.35	0.38
Lesion located in LAD	1.16	0.59-2.32	0.66			
Diameter stenosis	1.11†	0.92-1.35	0.44			
Reference diameter	0.87*∗*	0.45-1.71	0.69			
Lesion length	1.11†	0.70-1.76	0.64			

iFR, instantaneous wave-free ratio; FFR, fractional flow reserve; CI, confidence intervals; LAD, left anterior descending artery.

*∗*Per increase 1

†Per increase 10

‡Per increase 1000.

## Data Availability

The patients' clinical data used to support the findings of this study are restricted by the Tokyo Women's Medical University (TWMU) Ethics Committee in order to protect patient privacy. Data are available from clinical research support center TWMU for researchers who meet the criteria for access to confidential data. Researchers can contact corresponding author by e-mail.

## Supplementary Materials

Supplementary Materials Figure I: correlation between the rate-pressure product and iFR or FFR. Figure II: correlation between the cardiac index and iFR or FFR. Table I: hemodynamic factor according to iFR and FFR. Table II: univariate and multivariate analysis to evaluate predictors of discordance between iFR and FFR (limited to lesions of 40-80% stenosis).
