# Supplementary material for: Hemodynamic and Lesion Characteristics Associated with Discordance between the Instantaneous Wave-Free Ratio and Fractional Flow Reserve
Source: J Interv Cardiol. 2019 Jul 14;2019:3765282. doi: 10.1155/2019/3765282 (PMC6739793; doi:10.1155/2019/3765282)
Supplement: Supplementary Materials — Figure I: correlation between the rate-pressure product and iFR or FFR. Figure II: correlation between the cardiac index and iFR or FFR. Table I: hemodynamic factor according to iFR and FFR. Table II: univariate and multivariate analysis to evaluate predictors of discordance between iFR and FFR (limited to lesions of 40-80% stenosis). [file 3765282.f1.pdf]

Figure I (Data Supplement)

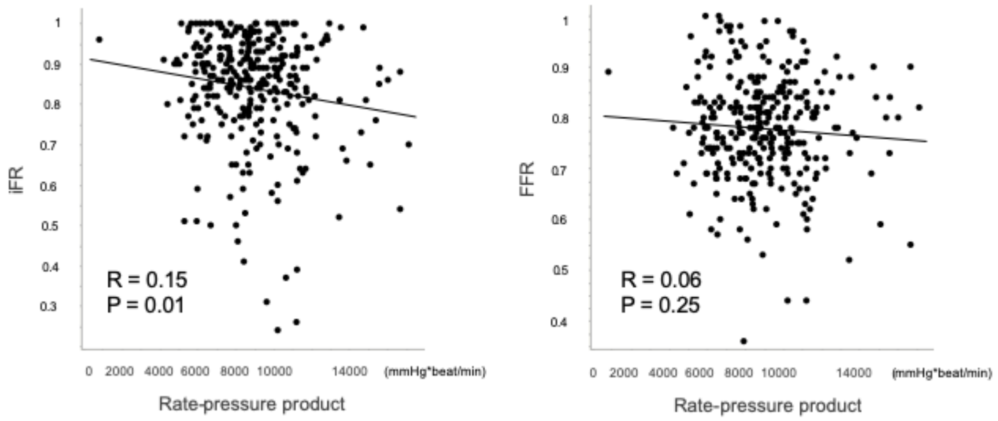

iFR: instantaneous wave-free ratio; FFR: fractional flow reserve

Figure II (Data Supplement)

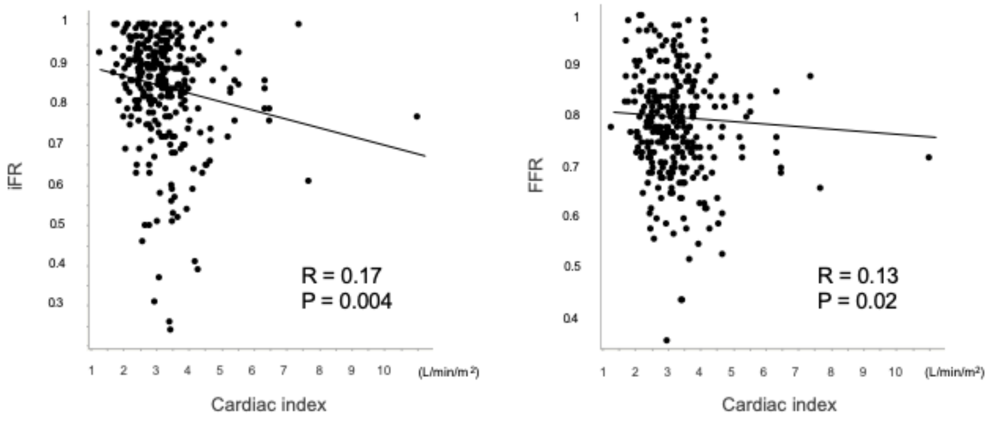

iFR: instantaneous wave-free ratio; FFR: fractional flow reserve

Table I. Hemodynamic factors according to iFR and FFR

	iFR ≦0.89 (n=181)	iFR >0.89 (n=123)	p-value
Systolic blood pressure, mmHg	125 ± 23	126 ± 25	0.71
Heart rate, beat/min	72 ± 12	71 ± 13	<0.01
Rate-pressure product, mmHg*beat/min	8967 ± 2337	9007 ± 2489	<0.01
Central vein pressure, mmHg	13.3 ± 5.6	12.2 ± 4.8	0.08
Pulmonary artery wedge pressure, mmHg	6.7 ± 3.0	6.6 ± 3.1	0.65
Cardiac index, L/min/m^2	3.4 ± 1.1	3.0 ± 0.8	<0.01
	FFR ≦0.8 (n=177)	FFR >0.8 (n=127)	p-value
Systolic blood pressure, mmHg	127 ± 23	123 ± 25	0.26
Heart rate, beat/min	74 ± 12	69 ± 12	0.83
Rate-pressure product, mmHg*beat/min	8967 ± 2337	9007 ± 2489	0.89
Central vein pressure, mmHg	6.7 ± 3.1	6.7 ± 3.0	0.99
Pulmonary artery wedge pressure, mmHg	13.0 ± 5.7	12.7 ± 4.8	0.63
Cardiac index, L/min/m^2	3.3±1.1	3.2±0.9	0.22

iFR, instantaneous wave-free ratio; FFR, fractional flow reserve.

Data are expressed as mean ± SD

Table II. Univariate and multivariate analysis to evaluate predictors of discordance between iFR and FFR (limited to lesions of 40-80 % stenosis)

A)

	Discordance of iFR ≦0.89 and FFR >0.8					
	Univariate analysis			Multivariate analysis		
	Odds ratio	95% CI	p-value	Odds ratio	95% CI	p-value
Age	1.04*	0.99-1.08	0.07	1.03*	0.98-1.07	0.24
Female sex	4.0	1.76-9.23	0.001	3.15	1.30-7.65	0.01
Diabetes mellitus	1.51	0.65-3.50	0.33			
Hypertension	1.10	0.42-2.86	0.86			
Hypercholesterolemia	1.71	0.66-4.43	0.27			
Hemodialysis	0.68	0.26-1.77	0.42			
Smoking	0.58	0.25-1.36	0.21			
Rate-pressure product	1.19‡	1.01-1.40	0.037	1.17‡	0.98-1.39	0.07
Cardiac index	1.03*	0.71-1.48	0.87			
Lesion located in LAD	0.69	0.31-1.53	0.37			
Diameter stenosis	1.10†	0.80-1.51	0.57			
Reference diameter	0.92*	0.43-1.96	0.83			
Lesion length	0.63†	0.33-1.19	0.13			

B)

	Discordance of iFR >0.89 and FFR ≦0.8					
	Univariate analysis			Multivariate analysis		
	Odds ratio	95% CI	p-value	Odds ratio	95% CI	p-value
Age	0.99*	0.96-1.03	0.77			
Female sex	0.74	0.27-2.05	0.56			
Diabetes mellitus	0.69	0.32-1.52	0.36			
Hypertension	0.74	0.31-1.79	0.51			
Hypercholesterolemia	1.44	0.58-3.55	0.42			
Hemodialysis	0.08	0.01-0.58	0.01	0.09	0.01-0.68	0.02
Smoking	1.27	0.58-2.77	0.55			
Rate-pressure product	0.77‡	0.63-0.95	0.01	0.79‡	0.63-0.99	0.04
Cardiac index	0.59*	0.35-1.00	0.03	0.86*	0.49-1.51	0.59
Lesion located in LAD	1.47	0.64-3.40	0.36			
Diameter stenosis	1.05†	0.77-1.45	0.75			
Reference diameter	0.97*	0.46-2.04	0.94			
Lesion length	1.16†	0.68-1.99	0.58			

iFR, instantaneous wave-free ratio; FFR, fractional flow reserve; CI, confidence intervals; LAD, left anterior descending artery.

*Per increase 1

†Per increase 10

‡Per increase 1000
